# Structure, content, unsafe abbreviations, and completeness of discharge summaries: A retrospective analysis in a University Hospital in Austria

**DOI:** 10.1111/jep.13533

**Published:** 2021-01-09

**Authors:** Christine Maria Schwarz, Magdalena Hoffmann, Christian Smolle, Michael Eiber, Bianca Stoiser, Gudrun Pregartner, Lars‐Peter Kamolz, Gerald Sendlhofer

**Affiliations:** ^1^ Research Unit for Safety in Health, c/o Division of Plastic, Aesthetic and Reconstructive Surgery, Department of Surgery Medical University of Graz Graz Austria; ^2^ Executive Department for Quality and Risk Management University Hospital Graz Graz Austria; ^3^ Division of Endocrinology and Diabetology, Department of Internal Medicine Medical University of Graz Graz Austria; ^4^ Department of Management, Health Management in Tourism University of Applied Sciences Bad Gleichenberg Austria; ^5^ Institute for Medical Informatics, Statistics und Documentation Medical University of Graz Graz Austria

**Keywords:** abbreviations, discharge summary, electronic health record, patient safety, risk

## Abstract

**Rationale and objective:**

The discharge summary (DS) is one of the most important instruments to transmit information to the treating general physician (GP). The objective of this study was to analyse important components of DS, structural characteristics as well as medical and general abbreviations.

**Method:**

One hundred randomly selected DS from five different clinics were evaluated by five independent reviewers regarding content, structure, abbreviations and conformity to the Austrian Electronic Health Records (ELGA) using a structured case report form. Abbreviations of all 100 DS were extracted. All items were scored on a 4‐point Likert‐type scale ranging from “strongly agree” to “strongly disagree” (or “not relevant”). Subsequently, the results were discussed among reviewers to achieve a consensus decision.

**Results:**

The mandatory fields, reason for admission and diagnosis at discharge were present in 80% and 98% of DS. The last medication was fully scored in 48% and the recommended medication in 94% of 100 DS. There were significant overall differences among clinics for nine mandatory items. In total, 750 unexplained abbreviations were found in 100 DS.

**Conclusions:**

In conclusion, DS are often lacking important items. Particularly important are a detailed medication history and recommendations for further medication that should always be listed in each DS. It is thus necessary to design and implement changes that improve the completeness of DS. An important quality improvement can be achieved by avoiding the use of ambiguous abbreviations.

**Abbreviations**
CRF: Case Report Form
DS: Discharge summary
ELGA: Electronic Health Record
eg: for example
GP: General practitioner
RECORD guidelines: Reporting of studies Conducted using Observational Routinely‐collected Data

## 1 INTRODUCTION

In order to ensure that patients are safely discharged from the hospital, the medical discharge summary (DS) represents one of the most important instruments to summarize all patient‐relevant medical information. Incomplete and inaccurate medical DS (important contents are not displayed, spelling mistakes, ambiguous wording, etc.) can lead to severe problems including an increase in the risk of re‐admission^1^, ^2^ and thus represent a barrier to efficient health services.^3^, ^4^, ^5^

Several issues related to the medical DS have already been identified.^6^ Delayed transmission of the DS to the further treating physician,^7^, ^8^, ^9^ low quality or lack of information,^10^, ^11^ lack of consistent formats,^12^, ^13^, ^14^ lack of patient understanding,^15^, ^16^ and inadequate training for medical students in writing medical DS^17^ are some important issues. The medical DS is not only an important document for the treating general physician (GP) but it is also relevant for other healthcare providers as well as patients and relatives.^18^ The use of specific medical jargon and unexplained abbreviations of medical terms hinder effective communication with all involved parties and cause relevant information to go unnoticed.^18^, ^19^, ^20^, ^21^, ^22^ According to Austrian law every patient must receive a DS at discharge and patients are owners of the written DS.^23^ In general, international studies have also reported that errors and unknown abbreviations in DS are often causing ambiguities.^21^, ^24^

DS with a summary in plain language support patients and relatives in understanding important information (eg, further recommended measures, medication intake).^25^

While it is mandatory in Austria to have a DS at discharge, there are currently no national standards regarding a unified structure. Every hospital is currently using a different structure which has often been criticized by GPs.

With the nation‐wide introduction of ELGA (Electronic Health Records), the Austrian digital documentation system in 2015, a standardized medical DS is required by law.^26^ However, although ELGA guidelines include mandatory headings a fully standardized DS is not yet adopted in practice by the Hospitals in Austria for many different reasons. The lack of standardization is leading to substantial variations even within one hospital. A standardization might improve DS practices and thus improve deficits in communication between hospitals and caregivers.

The aim of this study was to analyse medical DS at five different clinics at the University Hospital Graz, Styria, Austria, in order to assess the current implementation of the mandatory ELGA headings and structural items.

## 2 METHODS

### 2.1 Reporting

The research and reporting methodology followed the RECORD guidelines (“Reporting of studies Conducted using Observational Routinely‐collected Data”^27^) recommended by the EQUATOR network.

### 2.2 Sampling

Five different clinics (internal medicine, dermatology, surgery, neurology and paediatrics) at the University Hospital Graz were chosen to analyse and compare DS from different medical disciplines.

From February to September 2018, a total of 100 DS were collected by the Department of Finance and Controlling at the five different clinics. DS were collected on two randomly chosen days per week for patients hospitalized for 24 hours or longer. DS of patients who were transferred to another clinic before discharge were not considered. Five trained reviewers with backgrounds in medicine, nursing science, and quality and risk management independently analysed the DS. The 100 DS were a convenience sample out of all 120 discharges on those selected days.

### 2.3 Development of a Case Report Form (CRF)

A CRF was created to systematically review the DS (see Data S1). The items in the CRF were based on the results of a literature search in PubMed which identified a total of 209 key components of medical DS and the CRF also included the mandatory and optional headings of ELGA. The mandatory and optional headings of ELGA are presented in Table 1.

**TABLE 1:** Mandatory and optional ELGA headings of the medical DS

Option	Position	Section		
***[O]***	1	Letter text		
***[M]***	2	**Reason for admission**		Epicrisis
***[M]***	3	**Diagnosis at discharge**		
***[O]***	4	Rehabilitation goals		
***[O]***	5	Outcome Measurement		
***[O]***	6	Measures implemented		
***[M]***	7	**Last medication**		
***[M]***	8	**Recommended medication**		
***[M]***	9	**Further recommended measures**		
		***[R2]***	**Appointments, control**	
		***[R2]***	**Discharge condition**	
		***[R2]***	**Recommended arrangements for further care**	
***[O]***	10	Summary of stay		
***[O]***	11	Closing remarks		
***[R2]***	12	Allergies, intolerances and risks		Secondary Sections
***[O]***	13	Diagnostic findings Possible Subsections:		
		*[R2]*	Pending results	
		*[R2]*	Extracts from collected results	
		*[R2]*	Operation report	
		*[R2]*	Attached collected results	
		*[R2]*	Vital parameters	
***[O]***	14	Anamnesis		
***[O]***	15	Previous diseases		
		*[O]*	Subsection “Previous measures”	
***[O]***	16	Medication at admission		
***[O]***	17	Medication administered during the stay		
***[O]***	18	Living wills and other legal documents		
***[O]***	19	Supplements		

*Note*: MUST means a mandatory requirement (commandment). Corresponds to the conformity criteria [R] and [M]. SHOULD or RECOMMENDED stands for a recommendation. It is desired and recommended that the requirement should be implemented, but there may be reasons why this is not done. Corresponds to compliance criterion [R2]. CAN or OPTIONAL (MAY, OPTIONAL): The implementation of the requirement is optional, it can also be omitted without compelling reason. Corresponds to the conformity criterion [O].

The CRF was pre‐tested by several experts (nurses, physicians, and staff from the quality and risk management department) and all five reviewers, each using two DS (from different medical disciplines: surgery and internal medicine). With the pre‐test results, all reviewers were trained regarding the use of the CRF and the scoring using the Likert‐type scale.

### 2.4 Content of the CRF

The final CRF included 84 out of the 209 key components and was divided into different subsections such as structure and content, language specifications (general and medical abbreviations), typing errors, and length. Reviewers noted the presence of the items identified from literature and (mandatory and optional) ELGA headings (Data S1) and scored using the Likert‐type scale.

Each DS was reviewed by at least two independent reviewers. The reviewers individually scored each item on a 4‐point Likert‐type scale from “strongly agree” to “strongly disagree”, with the additional option of “not relevant”. After the individual scoring process, results were compared and discussed between the two reviewers. If there was a disagreement, a third reviewer was involved and the final scoring represents a consensus decision.

### 2.5 Abbreviations

All abbreviations and their frequencies of use were recorded for all 100 evaluated DS. Abbreviations were extracted by two additional independent reviewers. To integrate different spellings of the same abbreviation reviewers ignored lower/upper case, periods at the end of an abbreviation, removed blank spaces and replaced commas with periods (to distinguish commas used as a decimal separator).

### 2.6 Statistical analysis

Data was descriptively analysed using absolute and relative frequencies. Missing data and “not relevant” are explicitly displayed in the results. Fisher's exact test with a significance level of 0.05 was used to compare the medical disciplines. “Not relevant” scores were considered missing for these analyses. The analyses were performed using R version 3.6.1.^28^

## 3 RESULTS

In total, 100 DS from five clinics were evaluated: internal medicine (n = 30), dermatology (n = 20), surgery (n = 20), neurology (n = 8), and paediatrics (n = 22). The lengths of the 100 DS ranged from one to eight pages. Most DS (89%) were two to four pages long (two pages: 41 DS, three pages: 28 DS, four pages: 20 DS).

### 3.1 Use of mandatory ELGA headings

The mandatory items according to ELGA that have to be covered in the DS include: reason for admission, diagnosis at discharge, last/recommended medication, and further recommended measures. The reason for admission was scored as “strongly agree” in 80% of DS, and diagnosis at discharge was fully scored in 98% of DS. Further recommended measures were fully scored in 62% of the DS. Detailed results are presented in Table 2.

**TABLE 2:** Reporting of mandatory sections of ELGA in the sampled DS^a^

Section	1 = Strongly Agree	2 = Agree	3 = Disagree	4 = Strongly Disagree	Not applicable	Missing data
**Reason for admission**	80 (80%)	14 (14%)	6 (6%)			0
**Diagnosis at discharge**	98 (98%)	2 (2%)				0
**Last medication**	48 (48%)		1 (1%)	51 (51%)		0
Full name of the drug	35 (35.4%)	4 (4%)	3 (3%)	56 (56.6%)	1 (1%)	1
Dose or concentration of the drug	11 (11%)	4 (4%)	2 (2%)	82 (82%)	1 (1%)	0
Dosage form or method of application	13 (13%)	7 (7%)	7 (7%)	72 (72%)	1 (1%)	0
Frequency of administration	7 (7%)	2 (2%)	5 (5%)	85 (85%)	1 (1%)	0
**Recommended medication**	94 (94%)			6 (6%)		0
Full name of the drug	88 (88%)	5 (5%)	1 (1%)	6 (6%)		0
Dose or concentration of the drug	75 (75%)	15 (15%)	2 (2%)	8 (8%)		0
Dosage form or method of application	23 (23%)	17 (17%)	11 (11%)	49 (49%)		0
Frequency of administration	44 (44%)	44 (44%)	5 (5%)	7 (7%)		0
**Further recommended measures**	61 (62.2%)	14 (14.3%)	2 (2%)	21 (21.4%)		2
Appointments, control	66 (66.0%)	18 (18.0%)	8 (8.0%)	8 (8.0%)		0
Discharge condition	24 (24.0%)	45 (45.0%)	18 (18.0%)	13 (13.0%)		0
Recommended arrangements for further care	21 (21.2%)	28 (28.3%)	7 (7.1%)	43 (43.4%)		1

^**a**^ Data presented are numbers of observations, which coincide with percentages due to the total number of 100 DS. MUST means a mandatory requirement (commandment) [M].

According to ELGA, the “last medication” given in the hospital must be specified in case of a discharge to other hospitals or institutions, whereas the section “recommended medication” must be indicated in case of a discharge to a GP or specialist.

The last medication was scored fully (“strongly agree”) in 48% and the recommended medication in 94% of respective DS. The dose or concentration of the last medication was indicated in 11% of DS. Furthermore, the dosage form and method of administration was described in 13% of DS and the administration interval was presented in 7% of DS.

Regarding the recommended medication, the name of the drug was present in 88% of DS. The dose and concentration of the recommended medication was outlined in 75% of DS, whereas the dosage and method of administration was noted in 23% of DS. Detailed results see Table 2.

### 3.2 Differences between clinics

We examined differences among five medical disciplines regarding their use of mandatory DS headings. We found significant overall differences among clinics for nine mandatory headings: reason for admission (*P* < .001); last medication (*P* < .001); appointments, control (*P* = .002); discharge condition (*P* = .012); recommended arrangements for further care (*P* < .001); full name of the drug (last medication) (*P* < .001); frequency of administration (last medication) (*P* = .004); dosage form or method of application (recommended medication) (*P* < .001); frequency of administration (recommended medication) (*P* < .001). Statistically significant results are presented in Table 3 and all results are displayed in Data S2.

**TABLE 3:** Comparison of mandatory ELGA headings between five clinics

	Internal medicine (N = 30)	Dermatology (N = 20)	Surgery (N = 20)	Neurology (N = 8)	Paediatrics (N = 22)	Total (N = 100)	*P* value
Reason for admission							**<.001**
1 Strongly agree	30 (100.0%)	19 (95.0%)	2 (10.0%)	8 (100.0%)	21 (95.5%)	80 (80.0%)	
2 Agree	0 (0.0%)	1 (5.0%)	12 (60.0%)	0 (0.0%)	1 (4.5%)	14 (14.0%)	
3 Disagree	0 (0.0%)	0 (0.0%)	6 (30.0%)	0 (0.0%)	0 (0.0%)	6 (6.0%)	
Last medication							**<.001**
1 Strongly agree	21 (70.0%)	12 (60.0%)	0 (0.0%)	7 (87.5%)	8 (36.4%)	48 (48.0%)	
3 Disagree	1 (3.3%)	0 (0.0%)	0 (0.0%)	0 (0.0%)	0 (0.0%)	1 (1.0%)	
4 Strongly disagree	8 (26.7%)	8 (40.0%)	20 (100.0%)	1 (12.5%)	14 (63.6%)	51 (51.0%)	
Appointments, control							**.002**
1 Strongly agree	15 (50.0%)	16 (80.0%)	20 (100.0%)	2 (25.0%)	13 (59.1%)	66 (66.0%)	
2 Agree	6 (20.0%)	2 (10.0%)	0 (0.0%)	3 (37.5%)	7 (31.8%)	18 (18.0%)	
3 Disagree	4 (13.3%)	1 (5.0%)	0 (0.0%)	1 (12.5%)	2 (9.1%)	8 (8.0%)	
4 Strongly disagree	5 (16.7%)	1 (5.0%)	0 (0.0%)	2 (25.0%)	0 (0.0%)	8 (8.0%)	
Discharge condition							**.012**
1 Strongly agree	4 (13.3%)	8 (40.0%)	2 (10.0%)	1 (12.5%)	9 (40.9%)	24 (24.0%)	
2 Agree	15 (50.0%)	5 (25.0%)	10 (50.0%)	3 (37.5%)	12 (54.5%)	45 (45.0%)	
3 Disagree	4 (13.3%)	5 (25.0%)	6 (30.0%)	3 (37.5%)	0 (0.0%)	18 (18.0%)	
4 Strongly disagree	7 (23.3%)	2 (10.0%)	2 (10.0%)	1 (12.5%)	1 (4.5%)	13 (13.0%)	
Recommended arrangements for further care							**<.001**
N‐Miss	0	0	1	0	0	1	
1 Strongly agree	2 (6.7%)	6 (30.0%)	9 (47.4%)	1 (12.5%)	3 (13.6%)	21 (21.2%)	
2 Agree	2 (6.7%)	8 (40.0%)	10 (52.6%)	1 (12.5%)	7 (31.8%)	28 (28.3%)	
3 Disagree	3 (10.0%)	3 (15.0%)	0 (0.0%)	1 (12.5%)	0 (0.0%)	7 (7.1%)	
4 Strongly disagree	23 (76.7%)	3 (15.0%)	0 (0.0%)	5 (62.5%)	12 (54.5%)	43 (43.4%)	
Full name of the drug (last medication)							**<.001**
N‐Miss	0	1	1	0	0	2	
1 Strongly agree	13 (43.3%)	12 (63.2%)	0 (0.0%)	6 (75.0%)	4 (18.2%)	35 (35.7%)	
2 Agree	3 (10.0%)	0 (0.0%)	0 (0.0%)	1 (12.5%)	0 (0.0%)	4 (4.1%)	
3 Disagree	0 (0.0%)	0 (0.0%)	0 (0.0%)	0 (0.0%)	3 (13.6%)	3 (3.1%)	
4 Strongly disagree	14 (46.7%)	7 (36.8%)	19 (100.0%)	1 (12.5%)	15 (68.2%)	56 (57.1%)	
Frequency of administration (last medication)							**.004**
N‐Miss	0	1	0	0	0	1	
1 Strongly agree	1 (3.3%)	5 (26.3%)	0 (0.0%)	1 (12.5%)	0 (0.0%)	7 (7.1%)	
2 Agree	0 (0.0%)	2 (10.5%)	0 (0.0%)	0 (0.0%)	0 (0.0%)	2 (2.0%)	
3 Disagree	2 (6.7%)	2 (10.5%)	0 (0.0%)	0 (0.0%)	1 (4.5%)	5 (5.1%)	
4 Strongly disagree	27 (90.0%)	10 (52.6%)	20 (100.0%)	7 (87.5%)	21 (95.5%)	85 (85.9%)	
Dosage form or method of application (recommended medication)							**<.001**
1 Strongly agree	3 (10.0%)	2 (10.0%)	4 (20.0%)	3 (37.5%)	11 (50.0%)	23 (23.0%)	
2 Agree	7 (23.3%)	2 (10.0%)	1 (5.0%)	0 (0.0%)	7 (31.8%)	17 (17.0%)	
3 Disagree	2 (6.7%)	6 (30.0%)	2 (10.0%)	1 (12.5%)	0 (0.0%)	11 (11.0%)	
4 Strongly disagree	18 (60.0%)	10 (50.0%)	13 (65.0%)	4 (50.0%)	4 (18.2%)	49 (49.0%)	
Frequency of administration (recommended medication)							**<.001**
1 Strongly agree	4 (13.3%)	10 (50.0%)	16 (80.0%)	8 (100.0%)	6 (27.3%)	44 (44.0%)	
2 Agree	24 (80.0%)	7 (35.0%)	4 (20.0%)	0 (0.0%)	9 (40.9%)	44 (44.0%)	
3 Disagree	0 (0.0%)	0 (0.0%)	0 (0.0%)	0 (0.0%)	5 (22.7%)	5 (5.0%)	
4 Strongly disagree	2 (6.7%)	3 (15.0%)	0 (0.0%)	0 (0.0%)	2 (9.1%)	7 (7.0%)	

A post hoc analysis revealed which clinics differed significantly from each other regarding each mandatory heading (see Data S3).

### 3.3 Use of abbreviations in the DS

In total, 750 different abbreviations were found in the 100 evaluated DS. The 100 most common abbreviations are presented in Table 4.

**TABLE 4:** 100 most frequently used abbreviations found in the sampled DS^a^

Nr.	Abbreviation	Frequency	Nr.	Abbreviation	Frequency	Nr.	Abbreviation	Frequency
1	Z.N	79	36	VHFA	8	71	MAN	5
2	ST.P	66	37	AMB	7	72	NINS	5
3	BDS	42	38	BCC	7	73	PD	5
4	E	34	39	HB	7	74	PDA	5
5	TGL	30	40	MR	7	75	PLAST	5
6	AZ	29	41	MRT	7	76	RVOT	5
7	E‐NR	27	42	OS	7	77	S.C	5
8	PAT	22	43	RAPI	7	78	SHT	5
9	Z.B	20	44	SIN	7	79	TF	5
10	GTT	15	45	SR	7	80	ASDII	4
11	RE	14	46	AEZ	6	81	BA	4
12	STAT	14	47	AKT	6	82	CX	4
13	HF	13	48	CHRON	6	83	DEXT	4
14	LI	13	49	CT	6	84	ENTSPR	4
15	EZ	12	50	HBA1C	6	85	HT	4
16	HNO	12	51	I.E.L	6	86	I.V	4
17	MAX	12	52	LA	6	88	IT	4
18	RR	12	53	N	6	89	KC	4
19	V.A	12	54	O.B	6	90	KO	4
20	VA	12	55	TE	6	91	KU	4
21	IV	11	56	CCD‐MUXF3	5	92	LSF	4
22	V	11	57	CHIR	5	93	MEL	4
23	ART	10	58	CKD	5	94	NEG	4
24	BZW	9	59	DD	5	95	NSTEMI	4
25	COR	9	60	DG	5	96	OAD	4
26	HA	9	61	ED	5	97	PRÄP	4
27	SPO2	9	62	ERG	5	98	SEK	4
28	UE	9	63	GESLGE	5	99	SSW	4
29	US	9	64	GGF	5	100	TAPSE	4
30	AV	8	65	IAS	5			
31	CRP	8	66	INKL	5			
32	DIG	8	67	IVS	5			
33	DM	8	68	KHKIII	5			
34	DZT	8	69	LAD	5			
35	ECP	8	70	LT	5			

^a^ The following pre‐processing steps were carried out on the raw data as an attempt to catch and combine different spellings of the same abbreviation: lower/upper case was ignored, blank spaces were removed, points at the end of an abbreviation were ignored, commas were replaced by dots (to catch any commas used as a decimal separator).

## 4 DISCUSSION

### 4.1 Implications of findings

A complete and correct discharge information is crucial for patient safety and efficient health care provision after discharge.^29^, ^30^ Nevertheless, in practice, there are qualitative and quantitative differences in discharge documentation, which may affect patient safety and the understanding of patient‐related information. Other barriers at discharge that were reported by physicians, nurses, patients and relatives include low quality of information exchange, missing coordination of care, and a lack of communication between hospital and community care providers.^31^

### 4.2 Use of mandatory headings in sampled DS

Our analysis showed that some items of the DS had always higher scores than others. Mandatory fields (according to ELGA) such as reason for admission, diagnosis at discharge, and recommended medication were present in all 100 evaluated DS and content was largely complete. However, some optional yet important items, such as for example, details on medication, were often lacking.

Physicians previously agreed on including important items such as diagnosis (100%), therapy (99.7%), recommendations on further treatment (99.6%), prescription of medication (98.5%), as well as behavioural recommendations for patients (94.4%).^18^ This important information could be more easily structured and be immediately available with the implementation of electronic health records. Using electronic health records could also more easily improve the structural quality of DS, albeit not the use of abbreviations in text boxes and the resulting low comprehensibility.

### 4.3 Description of medication

We found that only few of the evaluated medical DS included specific details about the medication that was last given at the hospital or about further recommended medication. Accuracy and completeness of patients' medication information in the DS and notation of any changes are very important items to ensure patient safety and continuity of care. Previous studies found that as much 11% of patients' medication documentation showed discrepancies at discharge^32^ and that a quality improvement of DS resulted in fewer medication errors per patient.^10^ Since medication errors due to incomplete DS have the potential to cause serious harm to patients, the recommended medication after discharge is important to ensure that further treatment is safe and effective. The use of electronic DS has the potential to reduce discharge medication errors.^33^, ^34^, ^35^

### 4.4 Use of abbreviations in analysed DS

Generally, we found a lot of abbreviations in DS (750 abbreviations in 100 DS). Some of the most frequently used abbreviations are known to most physicians, but there was also a large number of very specific abbreviations that are probably not known to physicians from other specialties or GPs. Abbreviations are often considered an undesirable component of the DS as stated by 77.5% of physicians in a previous study,^18^ also because abbreviations are known to generate ambiguity such as for example in German DS, HA (an abbreviation that occurred nine times in the analysed DS) could mean “Hausarzt”, “Hepatitis A”, “Humanalbumin”, “Hämagglutinin” or “Hyaluronan”. Bechmann found that more than 50% of surveyed GPs stated that DS generally contain too many abbreviations, and 71% of the surveyed GPs felt that unknown abbreviations can usually not be deduced from the context. Nearly all respondents (94%) had to look up abbreviations either frequently or occasionally.^36^ Moreover, abbreviations are problematic for patients and relatives who often have difficulties even with standard medical abbreviations. Comprehensibility of DS for all subsequent users (physicians, home care, patients, etc.) could thus be greatly improved by a reduction or omission of abbreviations, in particular if those are ambiguous and not generally known.

Quality of the structure and completeness of content in a DS may also be affected by individual preferences of physicians, the medical training of the person writing or completing the DS, as well as the complexity of the patients' conditions and medications. Thus, courses in the medical curriculums that cover (correct) writing of medical DS are likely to raise the overall quality of DS. Feedback on written DS, the use of checklists and other well‐designed digital solutions may improve communication and have been shown to be successful methods in improving DS writing.^37^, ^38^, ^39^, ^40^, ^41^, ^42^

### 4.5 Strengths and limitations of the study

This present study highlights problems and deficiencies regarding the patient DS.

However, the study also has several limitations. Firstly, our analysis of existing DS was focused on the use of mandatory/optional structural items rather than the medical content. Secondly, we did not compare individual clinics in terms of number of used abbreviations because there was already high variation within each clinic regarding length and numbers of used DS.

## 5 CONCLUSION

In conclusion, DS are often lacking important items. Particularly important are a detailed medication history and recommendations for further medication that should always be listed in each DS. It is thus necessary to design and implement changes that improve the completeness of DS. An important quality improvement can be achieved by avoiding the use of ambiguous abbreviations.

## CONFLICT OF INTEREST

All authors declare that they have completed the declaration of interest outlined by the International Committee of Medical Journal Editors (ICMJE).

## AUTHOR CONTRIBUTIONS

C.M.S., M.H. guarantor. C.M.S., M.H., G.S. study concept and design. C.M.S, M.H., B.S., C.S., M.E. acquisition of data. C.M.S., M.H., G.P. analysis and interpretation of pooled data. C.M.S., M.H., G.S., L.P.K. drafting of the manuscript. All authors contributed for critical revision of the manuscript for important intellectual content. G.P., C.M.S., M.H. statistical analysis of pooled data.

## PATIENT CONSENT FOR PUBLICATION

Not required.

## ETHICS APPROVAL

The Ethics Committee of the Medical University of Graz approved the study (vote#: 29‐338 ex16/17).

## CONSENT FOR PUBLICATION

All authors confirmed the publication in its present form.

## TRANSPARENCY DECLARATION

The lead authors (C.M.S., M.H.) affirm that this manuscript is an honest, accurate, and transparent account of the study being reported; that no important aspects of the study have been omitted; and that any discrepancies from the study as planned have been explained.

## Supporting information


**Data S1.** Supporting Information.

**Data S2.** Supporting Information.

**Data S3.** Supporting Information.

## Data Availability

The datasets used and/or analysed during the current study are available from the corresponding author on reasonable request.
