# Supplementary material for: Structure, content, unsafe abbreviations, and completeness of discharge summaries: A retrospective analysis in a University Hospital in Austria
Source: J Eval Clin Pract. 2021 Jan 9;27(6):1243–51. doi: 10.1111/jep.13533 (PMC9290607; doi:10.1111/jep.13533)
Supplement: Supplementary file 1 — Data S1. Supporting Information. [file JEP-27-1243-s002.docx]

|  | | | | | | | | | | | | | | | | | | | | |
| --- | --- | --- | --- | --- | --- | --- | --- | --- | --- | --- | --- | --- | --- | --- | --- | --- | --- | --- | --- | --- |
| Bitte so markieren: □ X □ □ □ Bitte verwenden Sie einen Kugelschreiber oder nicht zu starken Filzstift. Dieser Fragebogen wird maschinell erfasst. | | | | | | | | | | | | | | | | | | | | |
| Korrektur: □ ■ □ X □ Bitte beachten Sie im Interesse einer optimalen Datenerfassung die links gegebenen Hinweise beim Ausfüllen. | | | | | | | | | | | | | | | | | | | | |
|  | | | | | | | | | | | | | | | | | | | | |
|  | | | | | | | | | | | | | | | | | | | | |
| 1. **Identifikationsdaten** | | | | | | | | | | | | | | | | | | | | |
| 1.1 **PrüferIn** | | | | | | | | | | | | | 1.3 Fall Nummer: | | | | | | | |
| □ BSBS □ CSS | | | | | | | | | | | | |  |  |  |  |  |  | | |
| □ MH □ ME | | | | | | | | | | | | |  | | | | | | | |
| □ NN | | | | | | | | | | | | |  | | | | | | | |
| 1.2 Datum der Analyse des Entlassungsbriefes | | | | | | | | | | | | |  | | | | | | | |
|  |  | |  | / |  |  | / |  |  |  |  |  |  | | | | | | | |
| 1.4 **Logo** der Institution (optional) | | | | | | | | | | | | | □ Ja | | | | | | □ Nein | □ nicht prüfbar |
| 1.5 **Name der Verfasserin/des Verfassers**  des Dokuments (optional) | | | | | | | | | | | | | □ Ja | | | | | | □ Nein | □ nicht prüfbar |
| 1.6 **Adresse der Verfasserin/des Verfassers**  des Dokuments (optional) | | | | | | | | | | | | | □ Ja | | | | | | □ Nein | □ nicht prüfbar |
| 1.7 **Funktion der Verfasserin/des Verfassers**  des Dokuments (optional) | | | | | | | | | | | | | □ Ja | | | | | | □ Nein | □ nicht prüfbar |
| 1.8 **Rechtliche/r UnterzeichnerIn** (muss) | | | | | | | | | | | | | □ Ja | | | | | | □ Nein | □ nicht prüfbar |
| 1.9 **Fachliche/r AnsprechpartnerIn** (muss) | | | | | | | | | | | | | □ Ja | | | | | | □ Nein | □ nicht prüfbar |
| 1.10 **Bemerkung:** | | | | | | | | | | | | |  | | | | | |  |  |
|  | | | | | | | | | | | | | | | | | | |  |  |

| 1.11 **Name der Organisation**, Klinik, Abteilung (optional) | □ Ja | □ Nein | □ nicht prüfbar |
| --- | --- | --- | --- |
| 1.12 **Adresse der Organisation**, Klinik, Abteilung  (optional) | □ Ja | □ Nein | □ nicht prüfbar |
| 1.13 **Universitätsklinik** für (optional) | □ Innere Medizin  □ Neurologie | □ Dermatologie  Und Venerologie  □ Kinder- und  Jugendheilkunde | □ Chirurgie |
| 1.14 **Andere** |  |  |  |

|  | | | | | | | | | | |  |
| --- | --- | --- | --- | --- | --- | --- | --- | --- | --- | --- | --- |
| 1.15 **Einweisende(r)/zuweisende(r)/überweisende**  **(r) Arzt/Ärztin** (R2) | | | | | □ Ja | | □ Nein | | | □ nicht prüfbar | |
| 1.16 **Name** der Empfängerin/des Empfängers (R2) | | | | | □ Ja | | □ Nein | | | □ nicht prüfbar | |
| 1.17 **Adresse** der Empfängerin/des Empfängers (R2) | | | | | □ Ja | | □ Nein | | | □ nicht prüfbar | |
| 1.18 **Funktion** der Empfängerin/des Empfängers (R2) | | | | | □ Ja | | □ Nein | | | □ nicht prüfbar | |
| 1.19 **Bemerkung:** | | | | |  | |  | | |  | |
|  | | | | | | | | | | |  |
| 1. **Identifikationsdaten** [Fortsetzung] | | | | | | | | | | | |
| 1.20 **Name** der Empfängerin/des Empfängers (R2) | | | | | □ Ja | | □ Nein | | | □ nicht prüfbar | |
| 1.21 **Name** der Patientin/des Patienten (optional) | | | | | □ Ja | | □ Nein | | | □ nicht prüfbar | |
| 1.22 **Adresse** der Patientin/des Patienten (optional) | | | | | □ Ja | | □ Nein | | | □ nicht prüfbar | |
| 1.23 **Geburtsdatum** der Patientin/des Patienten (optional) | | | | | □ Ja | | □ Nein | | | □ nicht prüfbar | |
| 1.24 **Versicherungsnummer** der Patientin/des  Patienten (optional) | | | | | □ Ja | | □ Nein | | | □ nicht prüfbar | |
| 1.25 **Bemerkung:** | | | | | | | | | | | |
|  | | | | | | | | |  |  |  |
| 1.26 Wird die/der **Patientin/der Patient im ärztlichen**  **Entlassungsbrief persönlich** adressiert? (optional) | | | | | □ Ja | | □ Nein | | | □ nicht prüfbar | |
| 1.27 **Datum** der Aufnahme (optional) | | | | | □ Ja | | □ Nein | | | □ nicht prüfbar | |
| 1.28 **Datum** der Entlassung oder Tod (optional) | | | | | □ Ja | | □ Nein | | | □ nicht prüfbar | |
| 1.29 **Datum** der Erstellung des ärztlichen  Entlassungsbriefes (optional) | | | | | □ Ja | | □ Nein | | | □ nicht prüfbar | |
| 1.30 **Bemerkung:** | | | | |  | |  | | |  | |
|  | | | | | | | | |  |  |  |
| 2. Epikrise | | | | | | | | | | | |
|  | |  | | Trifft voll zu | |  | |  | Trifft nicht zu | | nicht prüfbar |
| 2.1 | | **Aufnahmegrund**  (Grund der Einweisung, Beschreibung der Symptome, Verdachtsdiagnosen) (muss) | | □ | | □ | | □ | □ | | □ |
| 2.2 | | **Diagnose bei Entlassung**  (Auflistung der, während des Aufenthalts  erhobenen Diagnosen) (muss) | | □ | | □ | | □ | □ | | □ |
| 2.3 | | **Diagnosen** eingedeutscht? | | □ | | □ | | □ | □ | | □ |
| 2.4 | | **Rehabilitationsziele (bei Reha)**  ICF-orientiert formuliert (International  Classification of Functioning, Disability  and Health, Diagnosen) (optional) | | □ | | □ | | □ | □ | | □ |
| 2.5 | | **Outcome Measurement (bei Reha)** Das  Outcome Measurement oder die  medizinische Ergebnis-Messung erfolgt  mittels (indikationsspezifischer) Scores  und Lebensqualitätsfragebögen zu Beginn  und vor Ende des Reha-Aufenthaltes und  unterstützt die objektive Dokumentation  der Erreichung der Reha-Ziele. z.B.  Schmerz-Scala, FIM, etc. (optional) | | □ | | □ | | □ | □ | | □ |
| 2.6 | | **Bemerkung:** | |  | |  | |  |  | |  |
|  | | | | | | | | |  |  |  |
| 2. Epikrise [Fortsetzung] | | | | | | | | | | | |
|  | |  | | Trifft voll zu | |  | |  | Trifft nicht zu | | nicht prüfbar |
| 2.7 | | **Durchgeführte Therapie**  Kurzbeschreibung sämtlicher, während  des Aufenthalts durchgeführter  Therapiemaßnahmen, wie OPs, Eingriffe  oder sonstige Maßnahmen (optional) | | □ | | □ | | □ | □ | | □ |
| 2.8 | | **Bemerkung:** | |  | | | | | | | |
|  | | | | | | | | |  |  |  |
| 2.9 | | **PatientInnenverfügungen** und andere  juridische Dokumente (optional) | | □ | | □ | | □ | □ | | □ |
| 2.10 | | Vermerk **PatientInneninformationen/**  **Angehörigeninformationen.** Wurde  Patientin/Angehörige informiert? (optional) | | □ | | □ | | □ | □ | | □ |
| 2.11 | | Wurden der/dem PatientIn direkt  **Empfehlungen** für ihr/sein weiteres  persönliches gesundheitsförderndes  Verhalten gegeben? (z.B. Rauchstopp)  Informationen was mit PatientIn im Detail  besprochen wurde. (optional) | | □ | | □ | | □ | □ | | □ |
| 2.12 | | **Bemerkung:** | | □ | | □ | | □ | □ | | □ |
|  | | | | | | | | |  |  |  |
|  | | **Medikamente bei Einweisung** | | Trifft voll zu | |  | |  | Trifft nicht zu | | nicht prüfbar |
| 2.13 | | **Medikamente bei Einweisung** (optional) | | □ | | □ | | □ | □ | | □ |
| 2.14 | | **Vollständiger Name des Medikaments** (muss) | | □ | | □ | | □ | □ | | □ |
| 2.15 | | **Angabe des Wirkstoffes** (muss) | | □ | | □ | | □ | □ | | □ |
| 2.16 | | Werden zusätzlich zu Markennamen von Medikamenten oder Produkten **generische Namen** angegeben? | | □ | | □ | | □ | □ | | □ |
| 2.17 | | **Dosis oder Konzentration** des Medikamentes (muss) | | □ | | □ | | □ | □ | | □ |
| 2.18 | | **Darreichungsform** bzw. Applikationsart (muss) | | □ | | □ | | □ | □ | | □ |
| 2.19 | | **Darreichungsintervall** (muss) | | □ | | □ | | □ | □ | | □ |
| 2.20 | | **Zeitkritische Medikamente** (Angabe Zeitpunkt, wenn Zeitpunkt wichtig ist) (muss) | | □ | | □ | | □ | □ | | □ |
| 2.21 | | **Bedarfsmedikation (**optional) | | □ | | □ | | □ | □ | | □ |
| 2.22 | | **Bedarfsmedikation** mit spezifischer Indikation (optional) | | □ | | □ | | □ | □ | | □ |
| 2.23 | | **Hinweise ob Pat. „Mittel“ einnimmt die nicht ärztlich verordnet wird** (optional) | | □ | | □ | | □ | □ | | □ |
|  | | **Letzte Medikamente** | | Trifft voll zu | |  | |  | Trifft nicht zu | | nicht prüfbar |
| 2.24 | | **Letzte Medikation** (Die zuletzt im Spital gegebene Medikation) (muss) | | □ | | □ | | □ | □ | | □ |
| 2.25 | | Vollständiger **Name des Medikaments** (muss) | | □ | | □ | | □ | □ | | □ |
| 2.26 | | Angabe des **Wirkstoffes** (muss) | | □ | | □ | | □ | □ | | □ |
| 2.27 | | Werden zusätzlich zu Markennamen von Medikamenten oder Produkten **generische Namen** angegeben? | | □ | | □ | | □ | □ | | □ |
| 2.28 | | **Dosis oder Konzentration** des Medikamentes (muss) | | □ | | □ | | □ | □ | | □ |
| 2.29 | | **Darreichungsform** bzw. Applikationsart (muss) | | □ | | □ | | □ | □ | | □ |
| 2.30 | | **Darreichungsintervall** (muss) | | □ | | □ | | □ | □ | | □ |
| 2.31 | | **Bedarfsmedikation** (muss) | | □ | | □ | | □ | □ | | □ |
| 2.32 | | **Bedarfsmedikation** mit spezifischer Indikation (muss) | | □ | | □ | | □ | □ | | □ |
| 2.33 | | **Zeitkritische Medikamente** (Angabe Zeitpunkt, wenn Zeitpunkt wichtig ist) (muss) | | □ | | □ | | □ | □ | | □ |
| 2.34 | | **Bemerkung:** | |  | |  | |  |  | |  |
|  | | | | | | | | |  |  |  |

|  | | | | | | | | |
| --- | --- | --- | --- | --- | --- | --- | --- | --- |
|  | **Empfohlene Medikamente** | Trifft voll zu |  |  | Trifft nicht zu | nicht prüfbar |  |  |
| 2.35 | **Empfohlene Medikation** (empfohlene weitere Medikation nach der Entlassung) (muss) | □ | □ | □ | □ | □ |  |  |
| 2.36 | Vollständiger **Name des Medikaments** (muss) | □ | □ | □ | □ | □ |  |  |
| 2.37 | Angabe des **Wirkstoffes** (muss) | □ | □ | □ | □ | □ |  |  |
| 2.38 | Werden zusätzlich zu Markennamen von Medikamenten oder Produkten **generische Namen** angegeben? | □ | □ | □ | □ | □ |  |  |
| 2.39 | **Dosis oder Konzentration** des Medikamentes (muss) | □ | □ | □ | □ | □ |  |  |
| 2.40 | **Darreichungsform** bzw. Applikationsart (muss) | □ | □ | □ | □ | □ |  |  |
| 2.41 | **Darreichungsintervall** (muss) | □ | □ | □ | □ | □ |  |  |
| 2.42 | **Bedarfsmedikation** (muss) | □ | □ | □ | □ | □ |  |  |
| 2.43 | **Bedarfsmedikation** mit spezifischer Indikation (optional) | □ | □ | □ | □ | □ |  |  |

| 2. Epikrise [Fortsetzung] | | | | | | |
| --- | --- | --- | --- | --- | --- | --- |
| 2.44 | **Zeitkritische Medikamente** (Angabe Zeitpunkt, wenn Zeitpunkt wichtig ist) (muss) | □ | □ | □ | □ | □ |
| 2.45 | **Bemerkung:** |  | | | | |
|  | | | | | |  |

|  |  | Trifft voll zu |  |  | Trifft nicht zu | nicht prüfbar |
| --- | --- | --- | --- | --- | --- | --- |
| 2.46 | **Änderungen zur letzten Medikation (muss)** | □ | □ | □ | □ | □ |
| 2.47 | **Weitere empfohlene Maßnahmen** Therapieempfehlungen nach der Entlassung (außer Medikation) z. B. Anordnungen zum Wundmanagement, physikalische Therapien, Diätordnungen, Präventionsmaßnahmen, etc. (muss) | □ | □ | □ | □ | □ |
| 2.48 | **Weitere empfohlene Maßnahmen** als z.B. strukturierte Liste | □ | □ | □ | □ | □ |
| 2.49 | **Bemerkung:** |  |  |  |  |  |

|  |
| --- |

|  |  | Trifft voll zu |  |  | Trifft nicht zu | nicht prüfbar |
| --- | --- | --- | --- | --- | --- | --- |
| 2.50 | **Kontrolltermine** Auflistung weiterer Behandlungstermine, Kontrollen und Wiederbestellungen (R2) | □ | □ | □ | □ | □ |
| 2.51 | **Entlassungszustand** Informationen zur Belastbarkeit und Arbeitsunfähigkeit bzw. –fähigkeit, Aktivitäten des täglichen Lebens (R2) | □ | □ | □ | □ | □ |
| 2.52 | **Empfohlene Anordnungen an die weitere Pflege/Therapie** (Präzisierung der empfohlenen Delegation an die Berufsgruppe der der Pflege gemäß § 15 GuKG (R2)) | □ | □ | □ | □ | □ |
| 2.53 | **Zusammenfassung des Aufenthalts** Kurzbeschreibung des Verlaufs des stat. Aufenthalts (Dekurs) (optional) | □ | □ | □ | □ | □ |
| 2.54 | **Bemerkung:** |  |  |  |  |  |

|  | | | | | |  |  |
| --- | --- | --- | --- | --- | --- | --- | --- |
|  | | | | | |  |  |
| 3.sekundäre Sektionen | | | | | | | |
| 3.1 | **Allergien, Unverträglichkeiten und Risiken (R2)** | | Trifft voll zu |  |  | Trifft nicht zu | nicht prüfbar |
| 3.2 | **Ausstehende Befunde** (Hinweis) (ausstehende Befunde als Information für Dokumentempfängerin) (optional) | | □ | □ | □ | □ | □ |
| 3.3 | **Auszüge aus erhobenen Befunden** (zu jedem Auszug SOLL mindestens das Datum und die Art des Ursprungsbefundes angegeben werden) (optional) | | □ | □ | □ | □ | □ |
| 3.4 | **Operationsbericht** Informationen zu erfolgten Operationen für nachbehandelnde ÄrztInnen und andere GDA. (optional) | | □ | □ | □ | □ | □ |
| 3.5 | **Beigelegte erhobene Befunde**  Befunde die im Zuge des Aufenthalts erstellt wurden (optional) | | □ | □ | □ | □ | □ |
| 3.6 | **Vitalparameter** (z.B. Herzfrequenz, Blutdruck, Körpertemperatur, Sauerstoffsättigung) (optional) | | □ | □ | □ | □ | □ |
| 3.7 | **Anamnese**  (Gesundheitszustand der Patientin/des Patienten) (optional) | | □ | □ | □ | □ | □ |
| 3.8 | **Frühere Erkrankungen**  (Liste der bisherigen Krankheiten der Patientin/der Patienten) (optional) | | □ | □ | □ | □ | □ |
| 3.9 | **Bisherige Therapie**  Therapiemaßnahmen, die schon vor dem Aufenthalt durchgeführt wurden (optional) | | □ | □ | □ | □ | □ |
| 3.10 | **Bemerkung:** | |  |  |  |  |  |
|  | | | | | |  |  |

| 4. Inhalt | | | | | | |
| --- | --- | --- | --- | --- | --- | --- |
|  |  | Trifft voll zu |  |  | Trifft nicht zu | nicht prüfbar |
| 4.1 | Sind **Berichte von MTD oder Kliniksozialarbeit** enthalten? | □ | □ | □ | □ | □ |
| 4.2 | **Wenn ja, welche:** |  |  |  |  |  |
|  | | | | | | |
| 4.3 | Beschreibung,  wie bei **möglichen Komplikationen** gehandelt werden soll (optional) | □ | □ | □ | □ | □ |
| 4.4 | Beschreibung der **Vorsichtsmaßnahmen,** welche der Patient/die Patientin ergreifen soll | □ | □ | □ | □ | □ |
| 4.5 | **Kontaktdaten** für Krankenhausdienste (Sozialarbeiter/Innen, Psychologe, Psychologinnen, MTD…) | □ | □ | □ | □ | □ |
| 4.6 | **Quellen** für zuverlässige Informationen/Unterstützung (schriftliche Information, Selbsthilfe, Online) | □ | □ | □ | □ | □ |

| 5. Struktur + Sprache (Dekurs) | | | | | | | | | | |
| --- | --- | --- | --- | --- | --- | --- | --- | --- | --- | --- |
|  | |  | | Trifft voll zu |  | |  | Trifft nicht zu | | nicht prüfbar |
| 5.1 | | **Lesbarkeit** (subjektiver Eindruck) | | □ | □ | | □ | □ | | □ |
| 5.2 | | Verwendung **kurzer Sätze**  (durchschnittlich <15 Wörter im Dekurs) | | □ | □ | | □ | □ | | □ |
| 5.3 | | Informationen werden in einer logischen **Reihenfolge** dargestellt | | □ | □ | | □ | □ | | □ |
| 5.4 | | Das **Layout** Text ist thematisch unterteilt, Unterüberschriften bei Themenwechsel etc. sind vorhanden | | □ | □ | | □ | □ | | □ |
| 5.5 | | **Zahlen oder Grafiken** sind klar verständlich | | □ | □ | | □ | □ | | □ |
| 5.6 | | Anzahl aller **allgemeinen Abkürzungen** (im Dekurs) | | □ keine  □ 11 bis 20  □ >41 | | | □ bis 5  □ 21 bis 30 | | | □ 6 bis 10  □ 31 bis 40 |
| 5.7 | | **Inhaltlicher Umfang** (Seitenanzahl) | | □ 1  □ 4  □ 7 | | | □ 2  □ 5  □ >7 | | | □ 3  □ 6 |
| 5.8. | | Anzahl der **medizinischen Fachausdrücke** (im Dekurs) | | □ < 10  □ 31 bis 40 | | | □ 11 bis 20  □ >41 | | | □ 21 bis 30 |
| 5.9 | | **Anzahl der medizinischen Fachausdrücke** | | □ keine  □ 11 bis 15 | | | □ bis 5  □ mehr als 16 | | | □ 6 - 10 |
|  | |  | |  | | |  | | |  |
|  | |  | |  | | |  | | |  |
| 5. Struktur + Sprache (Dekurs) (Fortsetzung) | | | | | | | | | | |
| 5.10 | Anzahl der **Tippfehler (Dekurs)** | | □ keine  □ 11 bis 15 | | | □ bis 5  □ mehr als 16 | | | □ 6 - 10 | |
| 5.11 | **Bemerkung** | |  | | |  | | |  | |

|  |
| --- |
