# Supplementary material for: Structure, content, unsafe abbreviations, and completeness of discharge summaries: A retrospective analysis in a University Hospital in Austria
Source: J Eval Clin Pract. 2021 Jan 9;27(6):1243–51. doi: 10.1111/jep.13533 (PMC9290607; doi:10.1111/jep.13533)
Supplement: Supplementary file 2 — Data S2. Supporting Information. [file JEP-27-1243-s003.docx]

Supplemental Table 2 Comparison of mandatory ELGA headings

|  | Internal medicine (N=30) | Dermatology (N=20) | Surgery (N=20) | Neurology (N=8) | Pediatrics (N=22) | Total (N=100) | p value |
| --- | --- | --- | --- | --- | --- | --- | --- |
| Reason for admission |  |  |  |  |  |  | **< 0.001** |
| 1 Strongly agree | 30 (100.0%) | 19 (95.0%) | 2 (10.0%) | 8 (100.0%) | 21 (95.5%) | 80 (80.0%) |  |
| 2 Agree | 0 (0.0%) | 1 (5.0%) | 12 (60.0%) | 0 (0.0%) | 1 (4.5%) | 14 (14.0%) |  |
| 3 Disagree | 0 (0.0%) | 0 (0.0%) | 6 (30.0%) | 0 (0.0%) | 0 (0.0%) | 6 (6.0%) |  |
| Last medication |  |  |  |  |  |  | **< 0.001** |
| 1 Strongly agree | 21 (70.0%) | 12 (60.0%) | 0 (0.0%) | 7 (87.5%) | 8 (36.4%) | 48 (48.0%) |  |
| 3 Disagree | 1 (3.3%) | 0 (0.0%) | 0 (0.0%) | 0 (0.0%) | 0 (0.0%) | 1 (1.0%) |  |
| 4 Strongly Disagree | 8 (26.7%) | 8 (40.0%) | 20 (100.0%) | 1 (12.5%) | 14 (63.6%) | 51 (51.0%) |  |
| Diagnosis at discharge |  |  |  |  |  |  | 0.631 |
| 1 Strongly agree | 30 (100.0%) | 19 (95.0%) | 20 (100.0%) | 8 (100.0%) | 21 (95.5%) | 98 (98.0%) |  |
| 2 Agree | 0 (0.0%) | 1 (5.0%) | 0 (0.0%) | 0 (0.0%) | 1 (4.5%) | 2 (2.0%) |  |
| Recommended medication |  |  |  |  |  |  | 0.745 |
| 1 Strongly agree | 28 (93.3%) | 18 (90.0%) | 20 (100.0%) | 8 (100.0%) | 20 (90.9%) | 94 (94.0%) |  |
| 4 Strongly Disagree | 2 (6.7%) | 2 (10.0%) | 0 (0.0%) | 0 (0.0%) | 2 (9.1%) | 6 (6.0%) |  |
| Further recommended measures |  |  |  |  |  |  | 0.137 |
| N-Miss | 0 | 0 | 1 | 1 | 0 | 2 |  |
| 1 Strongly agree | 16 (53.3%) | 15 (75.0%) | 16 (84.2%) | 4 (57.1%) | 10 (45.5%) | 61 (62.2%) |  |
| 2 Agree | 5 (16.7%) | 2 (10.0%) | 3 (15.8%) | 1 (14.3%) | 3 (13.6%) | 14 (14.3%) |  |
| 3 Disagree | 1 (3.3%) | 0 (0.0%) | 0 (0.0%) | 0 (0.0%) | 1 (4.5%) | 2 (2.0%) |  |
| 4 Strongly Disagree | 8 (26.7%) | 3 (15.0%) | 0 (0.0%) | 2 (28.6%) | 8 (36.4%) | 21 (21.4%) |  |
| Appointments, control |  |  |  |  |  |  | **0.002** |
| 1 Strongly agree | 15 (50.0%) | 16 (80.0%) | 20 (100.0%) | 2 (25.0%) | 13 (59.1%) | 66 (66.0%) |  |
| 2 Agree | 6 (20.0%) | 2 (10.0%) | 0 (0.0%) | 3 (37.5%) | 7 (31.8%) | 18 (18.0%) |  |
| 3 Disagree | 4 (13.3%) | 1 (5.0%) | 0 (0.0%) | 1 (12.5%) | 2 (9.1%) | 8 (8.0%) |  |
| 4 Strongly Disagree | 5 (16.7%) | 1 (5.0%) | 0 (0.0%) | 2 (25.0%) | 0 (0.0%) | 8 (8.0%) |  |
| Discharge condition |  |  |  |  |  |  | **0.012** |
| 1 Strongly agree | 4 (13.3%) | 8 (40.0%) | 2 (10.0%) | 1 (12.5%) | 9 (40.9%) | 24 (24.0%) |  |
| 2 Agree | 15 (50.0%) | 5 (25.0%) | 10 (50.0%) | 3 (37.5%) | 12 (54.5%) | 45 (45.0%) |  |
| 3 Disagree | 4 (13.3%) | 5 (25.0%) | 6 (30.0%) | 3 (37.5%) | 0 (0.0%) | 18 (18.0%) |  |
| 4 Strongly Disagree | 7 (23.3%) | 2 (10.0%) | 2 (10.0%) | 1 (12.5%) | 1 (4.5%) | 13 (13.0%) |  |
| Recommended arrangements for further care |  |  |  |  |  |  | **< 0.001** |
| N-Miss | 0 | 0 | 1 | 0 | 0 | 1 |  |
| 1 Strongly agree | 2 (6.7%) | 6 (30.0%) | 9 (47.4%) | 1 (12.5%) | 3 (13.6%) | 21 (21.2%) |  |
| 2 Agree | 2 (6.7%) | 8 (40.0%) | 10 (52.6%) | 1 (12.5%) | 7 (31.8%) | 28 (28.3%) |  |
| 3 Disagree | 3 (10.0%) | 3 (15.0%) | 0 (0.0%) | 1 (12.5%) | 0 (0.0%) | 7 (7.1%) |  |
| 4 Strongly Disagree | 23 (76.7%) | 3 (15.0%) | 0 (0.0%) | 5 (62.5%) | 12 (54.5%) | 43 (43.4%) |  |
| Full name of the drug (last medication) |  |  |  |  |  |  | **< 0.001** |
| N-Miss | 0 | 1 | 1 | 0 | 0 | 2 |  |
| 1 Strongly agree | 13 (43.3%) | 12 (63.2%) | 0 (0.0%) | 6 (75.0%) | 4 (18.2%) | 35 (35.7%) |  |
| 2 Agree | 3 (10.0%) | 0 (0.0%) | 0 (0.0%) | 1 (12.5%) | 0 (0.0%) | 4 (4.1%) |  |
| 3 Disagree | 0 (0.0%) | 0 (0.0%) | 0 (0.0%) | 0 (0.0%) | 3 (13.6%) | 3 (3.1%) |  |
| 4 Strongly Disagree | 14 (46.7%) | 7 (36.8%) | 19 (100.0%) | 1 (12.5%) | 15 (68.2%) | 56 (57.1%) |  |
| Dose or concentration of the drug (last medication) |  |  |  |  |  |  | 0.052 |
| N-Miss | 0 | 1 | 0 | 0 | 0 | 1 |  |
| 1 Strongly agree | 2 (6.7%) | 5 (26.3%) | 0 (0.0%) | 2 (25.0%) | 2 (9.1%) | 11 (11.1%) |  |
| 2 Agree | 2 (6.7%) | 2 (10.5%) | 0 (0.0%) | 0 (0.0%) | 0 (0.0%) | 4 (4.0%) |  |
| 3 Disagree | 2 (6.7%) | 0 (0.0%) | 0 (0.0%) | 0 (0.0%) | 0 (0.0%) | 2 (2.0%) |  |
| 4 Strongly Disagree | 24 (80.0%) | 12 (63.2%) | 20 (100.0%) | 6 (75.0%) | 20 (90.9%) | 82 (82.8%) |  |
| Dosage form or method of application (last medication) |  |  |  |  |  |  | 0.064 |
| N-Miss | 0 | 1 | 0 | 0 | 0 | 1 |  |
| 1 Strongly agree | 3 (10.0%) | 5 (26.3%) | 0 (0.0%) | 1 (12.5%) | 4 (18.2%) | 13 (13.1%) |  |
| 2 Agree | 2 (6.7%) | 1 (5.3%) | 0 (0.0%) | 2 (25.0%) | 2 (9.1%) | 7 (7.1%) |  |
| 3 Disagree | 3 (10.0%) | 2 (10.5%) | 0 (0.0%) | 1 (12.5%) | 1 (4.5%) | 7 (7.1%) |  |
| 4 Strongly Disagree | 22 (73.3%) | 11 (57.9%) | 20 (100.0%) | 4 (50.0%) | 15 (68.2%) | 72 (72.7%) |  |
| Frequency of administration (last medication) |  |  |  |  |  |  | **0.004** |
| N-Miss | 0 | 1 | 0 | 0 | 0 | 1 |  |
| 1 Strongly agree | 1 (3.3%) | 5 (26.3%) | 0 (0.0%) | 1 (12.5%) | 0 (0.0%) | 7 (7.1%) |  |
| 2 Agree | 0 (0.0%) | 2 (10.5%) | 0 (0.0%) | 0 (0.0%) | 0 (0.0%) | 2 (2.0%) |  |
| 3 Disagree | 2 (6.7%) | 2 (10.5%) | 0 (0.0%) | 0 (0.0%) | 1 (4.5%) | 5 (5.1%) |  |
| 4 Strongly Disagree | 27 (90.0%) | 10 (52.6%) | 20 (100.0%) | 7 (87.5%) | 21 (95.5%) | 85 (85.9%) |  |
| Full name of the drug (recommended medication) |  |  |  |  |  |  | 0.297 |
| 1 Strongly agree | 28 (93.3%) | 16 (80.0%) | 19 (95.0%) | 7 (87.5%) | 18 (81.8%) | 88 (88.0%) |  |
| 2 Agree | 0 (0.0%) | 2 (10.0%) | 1 (5.0%) | 0 (0.0%) | 2 (9.1%) | 5 (5.0%) |  |
| 3 Disagree | 0 (0.0%) | 0 (0.0%) | 0 (0.0%) | 1 (12.5%) | 0 (0.0%) | 1 (1.0%) |  |
| 4 Strongly Disagree | 2 (6.7%) | 2 (10.0%) | 0 (0.0%) | 0 (0.0%) | 2 (9.1%) | 6 (6.0%) |  |
| Dose or concentration of the drug (recommended medication) |  |  |  |  |  |  | 0.152 |
| 1 Strongly agree | 25 (83.3%) | 11 (55.0%) | 18 (90.0%) | 6 (75.0%) | 15 (68.2%) | 75 (75.0%) |  |
| 2 Agree | 1 (3.3%) | 5 (25.0%) | 2 (10.0%) | 2 (25.0%) | 5 (22.7%) | 15 (15.0%) |  |
| 3 Disagree | 1 (3.3%) | 1 (5.0%) | 0 (0.0%) | 0 (0.0%) | 0 (0.0%) | 2 (2.0%) |  |
| 4 Strongly Disagree | 3 (10.0%) | 3 (15.0%) | 0 (0.0%) | 0 (0.0%) | 2 (9.1%) | 8 (8.0%) |  |
| Dosage form or method of application (recommended medication) |  |  |  |  |  |  | **< 0.001** |
| 1 Strongly agree | 3 (10.0%) | 2 (10.0%) | 4 (20.0%) | 3 (37.5%) | 11 (50.0%) | 23 (23.0%) |  |
| 2 Agree | 7 (23.3%) | 2 (10.0%) | 1 (5.0%) | 0 (0.0%) | 7 (31.8%) | 17 (17.0%) |  |
| 3 Disagree | 2 (6.7%) | 6 (30.0%) | 2 (10.0%) | 1 (12.5%) | 0 (0.0%) | 11 (11.0%) |  |
| 4 Strongly Disagree | 18 (60.0%) | 10 (50.0%) | 13 (65.0%) | 4 (50.0%) | 4 (18.2%) | 49 (49.0%) |  |
| Frequency of administration (recommended medication) |  |  |  |  |  |  | **< 0.001** |
| 1 Strongly agree | 4 (13.3%) | 10 (50.0%) | 16 (80.0%) | 8 (100.0%) | 6 (27.3%) | 44 (44.0%) |  |
| 2 Agree | 24 (80.0%) | 7 (35.0%) | 4 (20.0%) | 0 (0.0%) | 9 (40.9%) | 44 (44.0%) |  |
| 3 Disagree | 0 (0.0%) | 0 (0.0%) | 0 (0.0%) | 0 (0.0%) | 5 (22.7%) | 5 (5.0%) |  |
| 4 Strongly Disagree | 2 (6.7%) | 3 (15.0%) | 0 (0.0%) | 0 (0.0%) | 2 (9.1%) | 7 (7.0%) |  |
