# Supplementary material for: Structure, content, unsafe abbreviations, and completeness of discharge summaries: A retrospective analysis in a University Hospital in Austria
Source: J Eval Clin Pract. 2021 Jan 9;27(6):1243–51. doi: 10.1111/jep.13533 (PMC9290607; doi:10.1111/jep.13533)
Supplement: Supplementary file 3 — Data S3. Supporting Information. [file JEP-27-1243-s001.docx]

Supplemental Table 3 Post-hoc-Tests

| Variable | Internal medicine  vs.  Dermatology | Internal medicine  vs.  Surgery | Internal medicine  vs.  Neurology | Internal medicine  vs.  Pediatrics | Dermatology  vs.  Surgery | Dermatology  vs.  Neurology | Dermatology  vs.  Pediatrics | Surgery  vs.  Neurology | Surgery vs.  Pediatrics | Neurology vs.  Pediatrics |
| --- | --- | --- | --- | --- | --- | --- | --- | --- | --- | --- |
| Reason for admission | 0.400 | <0.001 | 1.000 | 0.423 | <0.001 | 1.000 | 1.000 | <0.001 | <0.001 | 1.000 |
| Last medication | 0.624 | <0.001 | 0.723 | 0.016 | <0.001 | 0.214 | 0.216 | <0.001 | 0.004 | 0.035 |
| Appointments, control | 0.258 | 0.001 | 0.570 | 0.206 | 0.106 | 0.030 | 0.229 | <0.001 | 0.003 | 0.074 |
| Discharge condition | 0.059 | 0.420 | 0.510 | 0.020 | 0.148 | 0.602 | 0.034 | 0.925 | 0.007 | 0.012 |
| Further recommended measures | <0.001 | <0.001 | 0.573 | 0.030 | 0.080 | 0.106 | 0.024 | <0.001 | <0.001 | 0.401 |
| Full name of drug (last medication) | 0.257 | <0.001 | 0.168 | 0.014 | <0.001 | 0.153 | 0.007 | <0.001 | 0.017 | 0.003 |
| Frequency of administration (Last medication) | 0.007 | 0.704 | 0.629 | 1.000 | <0.001 | 0.428 | 0.001 | 0.286 | 1.000 | 0.469 |
| Dosage form or method of application (recommended medication) | 0.148 | 0.316 | 0.103 | 0.001 | 0.413 | 0.389 | <0.001 | 0.707 | 0.001 | 0.047 |
| Frequency of administration (recommended medication) | 0.004 | <0.001 | <0.001 | 0.006 | 0.087 | 0.046 | 0.107 | 0.295 | 0.002 | 0.005 |

Legend: vs = versus

Note: Fisher’s exact test was used to compare the medical disciplines. Statistically significant: p= 0.005; Bonferroni correction.
